# Integrated genomics of ovarian xenograft tumor progression and chemotherapy response

**DOI:** 10.1186/1471-2407-11-308

**Published:** 2011-07-22

**Authors:** Ashley Stuckey, Andrew Fischer, Daniel H Miller, Sara Hillenmeyer, Kyu K Kim, Anna Ritz, Rakesh K Singh, Benjamin J Raphael, Laurent Brard, Alexander S Brodsky

**Affiliations:** 1 Molecular Therapeutics Laboratory, Program in Women's Oncology, Department of Obstetrics and Gynecology, Women and Infants7 Hospital, Alpert Medical School of Brown University, Providence, RI 02905, USA; 2 Department of Molecular Biology, Cell Biology, and Biochemistry, Brown University, Providence, RI 02903, USA; 3 Department of Computer Science & Center for Computational Molecular Biology, Brown University, Providence, RI 02912, USA; 4 Department of Obstetrics and Gynecology, Division of Gynecologic Oncology, Southern Illinois University School of Medicine, Springfield, IL 62794-9640, USA

## Abstract

**Background:**

Ovarian cancer is the most deadly gynecological cancer with a very poor prognosis. Xenograft mouse models have proven to be one very useful tool in testing candidate therapeutic agents and gene function *in vivo*. In this study we identify genes and gene networks important for the efficacy of a pre-clinical anti-tumor therapeutic, MT19c.

**Methods:**

In order to understand how ovarian xenograft tumors may be growing and responding to anti-tumor therapeutics, we used genome-wide mRNA expression and DNA copy number measurements to identify key genes and pathways that may be critical for SKOV-3 xenograft tumor progression. We compared SKOV-3 xenografts treated with the ergocalciferol derived, MT19c, to untreated tumors collected at multiple time points. Cell viability assays were used to test the function of the PPARγ agonist, Rosiglitazone, on SKOV-3 cell growth.

**Results:**

These data indicate that a number of known survival and growth pathways including Notch signaling and general apoptosis factors are differentially expressed in treated vs. untreated xenografts. As tumors grow, cell cycle and DNA replication genes show increased expression, consistent with faster growth. The steroid nuclear receptor, PPARγ, was significantly up-regulated in MT19c treated xenografts. Surprisingly, stimulation of PPARγ with Rosiglitazone reduced the efficacy of MT19c and cisplatin suggesting that PPARγ is regulating a survival pathway in SKOV-3 cells. To identify which genes may be important for tumor growth and treatment response, we observed that MT19c down-regulates some high copy number genes and stimulates expression of some low copy number genes suggesting that these genes are particularly important for SKOV-3 xenograft growth and survival.

**Conclusions:**

We have characterized the time dependent responses of ovarian xenograft tumors to the vitamin D analog, MT19c. Our results suggest that PPARγ promotes survival for some ovarian tumor cells. We propose that a combination of regulated expression and copy number can identify genes that are likely important for chemotherapy response. Our findings suggest a new approach to identify candidate genes that are critical for anti-tumor therapy.

## Background

Epithelial ovarian cancer (EOC) is the most lethal of all the gynecologic cancers, affecting thousands of women each year [1]. Most patients initially respond to chemotherapy, only to recur within a few years with drug-resistant metastatic disease [2]. Thus, there is a pressing need to develop new anti-tumor therapies that can work alone, or in combination with platinum-based therapy.

Two general approaches have been pursued to address drug resistance: development of new therapeutics, and drug combinations that improve standard platinum and/or taxane based chemotherapy. The application of calcitriol/vitamin D3 has emerged as an important strategy to target the vitamin D receptor (VDR) for cancer treatment [3]. Hypercalcemia and other toxicities have limited development of calcitriol and vitamin D analogs tested to date [3].

MT19c is a novel vitamin D analog based on B3CD [4,5] that shows significant effects on EOC cell lines and xenograft tumor models. MT19c was designed to be a vitamin D receptor ligand but appears to work independently of VDR (Brard L, Lange TS, Robinson K, Kim KK, Brodsky AS, Uzun A, Padbury J, Moore R, Singh RK: Discovery of the first Ergocalciferol derived vitamin D receptor independent true non-hypercalcemic anti-cancer agent (MT19c), *submitted*). Here, we aimed to understand which pathways and genes may be important for MT19c action in a SKOV-3 xenograft tumor model. These data also provide insight into key pathways and genes important for tumor growth and survival.

As EOC progresses, tumors may evolve through two general mechanisms: accumulation of new mutations, or selection of specific cell types emerging from a heterogeneous mixture of cells [6]. In the clinic, examination of tumors is typically only feasible as a snapshot at a given time with little knowledge about how a tumor is evolving during disease progression. A recent evaluation of long-term platinum treatment of a mouse lung cancer model suggested that DNA repair pathways are significantly up-regulated leading to resistance [7].

Many mutations and chromosomal structural rearrangements have been identified in primary ovarian tumors and cell lines [8-10]. Copy number aberrations (CNAs) are a common mechanism observed to control gene expression and tumor progression [8]. Loss of DNA is another mechanism that reduces expression of tumor suppressor genes, which inhibit tumor progression. Conversely, DNA copy number gain may increase expression of oncogenes. However, CNAs can explain a significant fraction of the variation in gene expression but not all of it, perhaps because of epigenetic mechanisms such as DNA methylation [11,12].

The purpose of this study was to understand which genes and pathways may be important for MT19c's anti-tumor activity and to identify genes critical for tumor progression. A number of genes in the PPARγ network, including PPARγ, were enriched in MT19c treated tumors. When PPARγ is stimulated with Rosiglitazone, MT19c and cisplatin have significantly higher IC_50_s suggesting that PPARγ is promoting survival in at least some types of ovarian cancer cells, leading to poorer outcomes. By combining CNAs and drug induced expression changes, we observe a subset of genes that may be particularly important for MT19c action and/or tumor survival. We propose that combining copy number analysis with drug induced expression changes can identify key genes important for chemotherapeutic efficacy. These results will be relevant to plan future xenograft studies and highlight the importance of considering the changing tumor dynamics over time when evaluating gene expression and drug responses.

## Methods

### Cell culture and Reagents

SKOV-3 (ATCC) cells were grown DMEM (Mediatech, Manassas, VA) with 10% FBS (Hyclone, Logan, UT) with. MT19c was synthesized as described in detail elsewhere [13]. Briefly, commercially available Ergocalciferol underwent a Diels-Alder reaction with N-methyl,1,2,4-triazolinedione to generate an adduct, which upon reaction with bromoacetic acid in the presence of DCC (N, N'-dicyclohexylcarbodiimide) generated MT19c in good yields. Rosiglitazone was purchased from Axxora (San Diego, CA). GW9662 was purchased from Sigma-Aldrich (St Louis, MO).

### SKOV-3 Xenograft Tumors and Treatment

SKOV-3 cells (2 × 10^6 ^cells/inoculate) were suspended in 0.1 mL of matrigel and inoculated subcutaneously in the flank of 4-6 week-old nude mice (NU/NU; strain code 088/homozygous) (Charles River Laboratories, Wilmington, MA) two weeks before treatment. MT19c was prepared as a stock solution of 1 mM in 100% EtOH and diluted 1:40 in PBS for administration. Mice were monitored and treated intraperitoneally (IP) every other day with either vehicle control (control group, PBS/2.5% EtOH; 12 animals) or 0.3 mL (10 mg/kg bwt) of MT19c (10 animals). Tumor size was calculated using a caliper. Animal experiments were carried out in the animal facilities of Rhode Island Hospital (RIH), RI, USA with strict adherence to the guidelines of the Animal Welfare Committee of RIH and Women and Infants Hospital (IACUC protocol # 0061-07).

### RNA purification and microarrays

RNA was isolated from each tumor by homogenization in Trizol (Invitrogen) using a Tissuemizer (Tekmar Company, Cincinnati, OH). RNA was purified using Qiagen RNAsy columns following manufacturer's protocol. All RNA was of high quality as assessed by an Agilent 2100 Bioanalyzer (Agilent Technologies, Inc. Santa Clara, CA). RNA was prepared for hybridization on Affymetrix Human Gene 1.0 St arrays. All RNA processing, array hybridizations and scanning were performed by the Brown University Center for Genomics and Proteomics core facility. RNA was prepared for hybridization using Affymetrix standard protocols and applied to Human Gene 1.0 ST microarrays (Affymetrix, Santa Clara, CA). All microarrays were quantile normalized together and the Probe Logarithmic Intensity Error (PLIER) algorithm was used to generate signal estimates for all RefSeq genes. To select actual signal, we discarded those transcripts belonging to the lowest quartile of their respective datasets. Analysis of significantly changing genes was determined using R http://www.r-project.org/. Complete microarray data have been deposited at the National Center for Biotechnology Information's Gene Expression Omnibus (accession number GSE23616).

### Ingenuity Pathway Analysis

The discriminating genes were used as input into Ingenuity Pathway Analysis (IPA) (Ingenuity IP 8.6-3003 http://www.ingenuity.com. The following analysis settings were used, the Ingenuity Knowledge database for genes on the Affymetrix Human Gene 1.0 St Array was used as a reference set, direct and indirect relationships were included, with a maximum of 35 molecules per network and a maximum of 25 networks per analysis. All data sources and species, and all tissues and cell lines were included. IPA uses a Fischer's exact test to determine which pathways and biological functions are significantly enriched in the input gene set relative to the reference set.

### Quantitative Real-Time PCR

Equal amounts of total RNA were reverse transcribed using Superscript III and random hexamers (Invitrogen, Carlsbad, CA). Resulting cDNA was renormalized using Quant-iT PicoGreen (Invitrogen) before mixing with 1× Power SYBR Green PCR Master Mix (Applied Biosystems, Foster City, CA). Reactions were performed in an Applied Biosystems 7900HT Fast Real-Time PCR System. The fold change was calculated as described previously using calnexin as an internal control. Primer sequences are listed in Additional file 1 Table S1.

### DNA purification and CGH arrays

**Figure 1:**
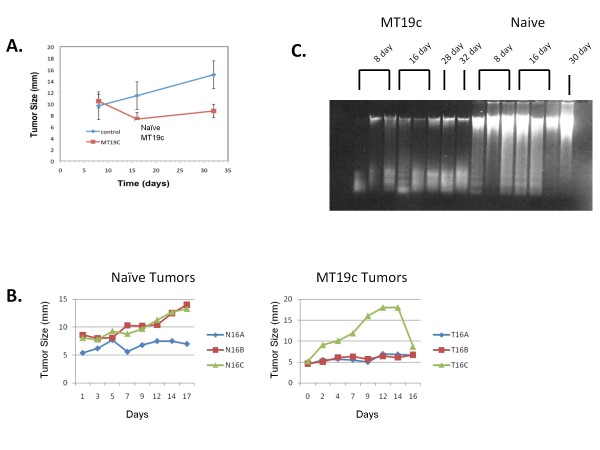
**MT19c reduces growth of SKOV-3 xenograft tumors and induces apoptosis**. **A**. MT19c significantly reduced the growth of xenograft tumors on average. The size of each tumor was measured with calipers, average for each time point and plotted. Error bars are standard deviations of the mean tumor measurements. **B**. Individual growth curves for the naïve (N16A, B, C) and MT19c treated (T16A, B, C) xenograft tumors **C**. Purified DNA reveals MT19c induced degradation of DNA. One μg of DNA from each specimen was purified and run on an agarose gel. All MT19c treated tumors had significantly degraded DNA while the majority of the DNA from naïve tumors is in a distinct upper band indicative of high molecular weight DNA.

DNA was isolated using Qiagen DNasy Blood and Tissue Kit following manufacturer's instructions. DNA quality was determined on an agarose gel as shown in Figure 1. DNA was prepared for Agilent 180 K CGH microarrays using the Roche NimblGen (Madison, WI) enzymatic labeling protocol using random nonamers and hybridized following manufacturer's protocols at the Microarray Centre at the Prostate Centre in Vancouver, British Columbia, Canada. Each sample was hybridized with Promega (Madison, WI) female reference DNA. The log10 copy number ratios were smoothed using a standard deviation-based outlier detection method and segmented using Circular Binary Segmentation (CBS) [14] as implemented in the R package DNAcopy ('smooth.region = 10' for the smoothing method, 'alpha = 0.05' for the segmentation, and default parameters for all other arguments). A log10 copy number for each gene was computed by averaging the smoothed and segmented log10 ratios for each probe located in within the gene region. Only genes that contained three or more probes were considered.

### Cell viability measurements

Cells were plated in 96-well plates and, 24 hours later, were treated with the indicated combinations of compounds. Viability was measured after 96 hours by WST-1 (Roche). All assays were done in biological triplicate, with technical triplicates done for each biological repeat.

## Results

### Experimental design

To understand the genes and pathways that drive tumor growth and drug response, we compared MT19c and vehicle treated SKOV-3 xenografts in nude mice. SKOV-3 expresses VDR (Additional file 2 Figure S1) and is a common cell line to model EOC. We aimed to understand how a tumor evolves during treatment compared to untreated, growing tumors. Measurements of tumor size revealed that MT19c significantly reduced tumor size or limited growth (Figure 1A and 1B). Past experiments with larger numbers of mice show more statistically significant MT19c effects [15,16]. MT19c induced DNA degradation, consistent with apoptosis, further demonstrating MT19c's anti-tumor activity (Figure 1C). MT19c is known to induce apoptosis in the SKOV-3 cell line [15,16]. Xenograft tumors were collected at multiple time points after the initiation of treatment as indicated by the labels where the first letter indicates the treatment (T for MT19c and N for naïve), the number indicates the number of days, and the final letter indicates the replicate. For example, T8A indicates the A replicate of an 8 day MT19c treated tumor.

The T16C xenograft tumor has a notably different MT19c response compared to the other tumors. T16C appeared to grow before rapidly shrinking. Upon collection, T16C appeared to have a liquid center, which may indicate necrosis. The DNA from T16C was significantly degraded as shown in Figure 1C. Thus, MT19c appears to repress the T16C xenograft tumor effectively but differently than the other tumors. The naïve tumor, N16A, appeared not to have grown significantly during the experiment. However, clustering and other expression behaviors indicate that this xenograft tumor is more similar to the other naïve tumors compared to the MT19c treated tumors. In addition, H&E staining indicated that each xenograft tumor was not significantly infiltrated with vascularization (Additional file 3 Figure S2). Together, these observations suggest that MT19c is an effective anti-tumor molecule and that the majority of the purified nucleic acids were derived from human tumor cells in this xenograft system.

### MT19c induced gene expression changes

To identify genes and pathways that are differentially expressed upon MT19c treatment and tumor progression, we took a genome-wide approach. RNA was purified from each tumor and probed with Affymetrix Human Gene St 1.0 microarrays.

**Figure 2:**
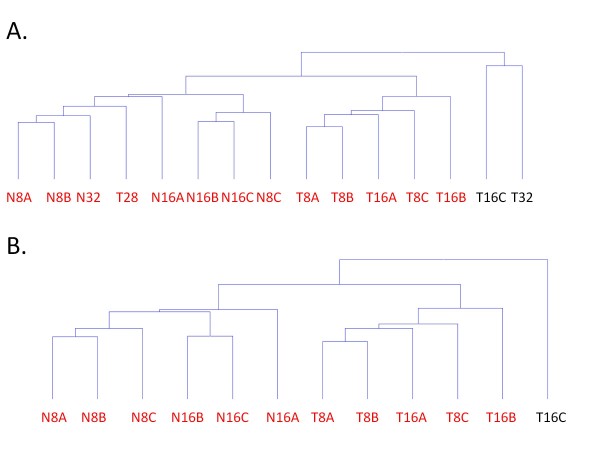
**Hierarchical clustering of mRNA expression in each tumor suggests MT19c treatment is the defining feature of the panel of xenograft tumors**. **A**. All samples were clustered by HCE Hierarchical Clustering Explorer revealing four major groups. Outliers are in black. **B**. Tumors collected after 16 days were removed from the clustering revealing two distinct classes defined by MT19c treatment.

To determine which tumors are most alike, the top 75% of all expressed genes were hierarchically clustered using Hierarchical Clustering Explorer (HCE). Tumors collected after treatment initiation do not clearly separate by time. Figure 2A shows that the two longer treatment time points, T28 and T32, are in the same clusters with the other time points suggesting that time was not the defining factor. However, the tumors can be classified into distinct clusters based on treatment when the long time points, T28 and T32, are removed (Figure 2B). The treated and control tumors cluster together except for T16C which appears to be an outlier in its expression profile, growth curve and was very necrotic, with a liquid middle not apparent in the other tumors T16C also had a distinct growth profile, highlighted by rapid increase followed by a sharp decrease in tumor size (Figure 1B). We therefore thought it reasonable to exclude T16C to identify key genes involved in MT19c action.

Because the tumors collected at longer times appear to be outliers from the clustering analysis, we focused on the first two time points to assess treatment-dependent and time-dependent changes. To gain insight into consistent MT19c induced effects, we compared the treated and naïve tumors from the first two time points, days 8 and 16, excluding the T16C outlier. We identified the most significantly MT19c regulated transcripts by a t-test (q < 0.05) and log2 fold change > 0.6 (Additional files 4 and 5, Tables S2 and S3). MT19c up-regulated 268 and down-regulated 306 genes when comparing the six naïve tumors from the 8 day and 16 day time points to the five MT19c treated tumors from the same time points that clustered similarly (T8A, T8B, T8C, T16A, T16B) (Figure 2). To validate the data, we sampled genes by real-time qPCR and found good concordance in trend but not magnitude (Additional file 6 Figure S3). This is a common observation when comparing microarray and qPCR data, where the microarray dynamic range is reduced compared to qPCR [17].

### MT19c regulated pathways

**Figure 3:**
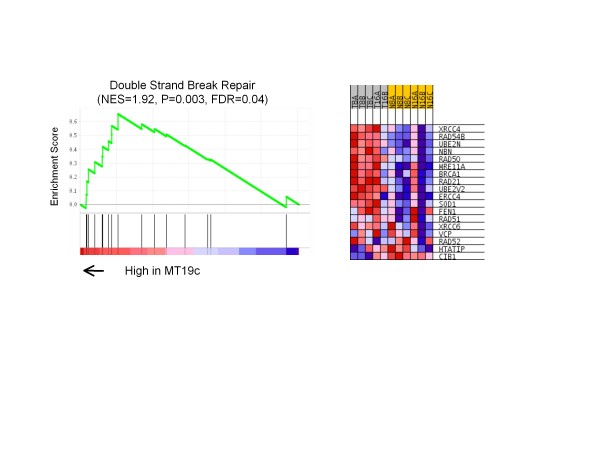
**Genes associated with double strand break repair are up-regulated by MT19c**. Enrichment plot of the double strand break repair gene set identified by GSEA and corresponding heat map for MT19c and naïve tumors. Expression level is represented as a gradient from high (red) to low (blue).

To gain insight into MT19c regulated pathways, we used Gene Set Enrichment Analysis (GSEA) to identify enriched pathways mediated by MT19c and tumor progression over time. GSEA uses all Refseq transcripts on the microarray data to identify biases in tested pathways between two conditions [18]. Table 1 summarizes pathways enriched in treated vs. naïve tumors. Energy metabolism genes including transcripts coding for oxidative phosphorylation machinery were down-regulated by MT19c compared to naïve tumors. These metabolic pathways may be higher in the naïve tumors because these tumors may have been growing faster and were therefore more metabolically active. On the other hand, genes involved in double strand break repair, protein turnover, and apoptosis were enriched in MT19c treated tumors. The double strand break repair pathway is highlighted by stimulation of XRCC4, RAD54B and RAD50 as show in Figure 3. These protein products are thought to directly repair DNA and not just be part of a general stress response. Stimulation of DNA repair pathways may help tumor cells survive the lethal effects of MT19c or, alternatively, MT19c may be inducing DNA damage to elicit this response. Grouping the MT19c treated and naïve tumors identified numerous pathways and genes differentially regulated.

**Table 1:** Gene Set Enrichment Analysis reveals MT19C down-regulation of energy metabolism and stimulation of DNA repair and apoptosis pathways.

Geneset	NES	P-value	FDR GSEA
**Kegg (n = 165)**			
Cell Communication	-1.97	0.000	0.029
Biosynthesis of Steroids	-1.95	0.002	0.046
Galactose metabolism	-1.84	0.005	0.082
Oxidative Phosphorylation	-1.69	0.000	0.102
Notch signaling pathway	-1.49	0.032	0.232
Ubiquitin mediated Proteolysis	1.98	0.000	0.021
			
**Gene Ontology (n = 1307)**			
Double Strand Break Repair	1.86 (1.85)	0.000 (0.006)	0.006
Ubiquitin Cycle	2.01 (1.96)	0.000 (0.000)	0.111

NES > 1.4 indicates enriched in MT19C treated tumors compared to naïve tumors for all 8 and 16 day tumors, including T16C.

Specific time dependent changes may be observed by examining each time point separately as opposed to grouping the treated and naïve tumors. Comparing day 8 treated to the day 8 naïve tumors, we observed down-regulation of genes in cell cycle control, DNA replication, and up-regulation of energy metabolism genes (Table 2). On the other hand, comparing day 16 treated and naïve tumors revealed up-regulation of protein synthesis machinery (Table 3). These data indicate that MT19c initially affects tumor growth. The cell cycle pathway enriched in day 8 treated vs. day 8 naïve tumors is highlighted by CCNE2, a cyclin that peaks during G1-S phase transition [19]. This is consistent with the MT19c induced cell cycle arrest [13]. At later times, translation factors and protein synthesis machinery are up-regulated by MT19c perhaps reflecting the tumor's attempts to try to survive by up-regulating other genes at the translational level.

**Table 2:** Increased expression of cell cycle regulators as during naïve tumor growth by GSEA.

Geneset	NES	P-value	FDR GSEA
**Naïve Tumor 8 day vs. 16 day**			
**BioCarta (n = 184)**			
Cell cycle pathway	-2.06	0.000	0.065
**Kegg (n = 165)**			
Ribosome	2.25	0.000	0.000
Cell Cycle	-2.34	0.000	0.000
Proteasome	-1.72	0.000	0.088
**GenMapp (n = 107)**			
DNA replication reactome	-2.13	0.000	0.003
Cholesterol Biosynthesis	-1.89	0.012	0.024
**TFT (n = 614)**			
E2F_Q3	-1.84	0.000	0.039
E2F1_Q6	-1.76	0.000	0.027
**Gene Ontology (n = 1307)**			
M phase of mitotic cell cycle	-2.15	0.000	0.008
M phase	-2.17	0.000	0.007
Mitosis	-2.18	0.000	0.008
			
***MT19C 8 day vs. 16 day***			
**GenMapp (n = 107)**			
DNA Replication Reactome	1.74	0.000	0.056
Ribosomal Proteins	-2.44	0.000	0.000
Proteasome	-1.76	0.016	0.091
**Kegg (n = 165)**			
Cell Cycle	1.68	0.004	
Apoptosis	-1.54	0.016	0.164
**Transcription Factor Targets (n = 614)**			
E2F_Q4	1.83	0.000	0.025
E2F1_Q6	1.77	0.000	0.018
NFKB	-1.79	0.000	0.021
AP1	-1.78	0.000	0.024
NFKAPPAB	-1.52	0.000	0.063

NES > 1.4 indicates higher expression in 8 day naïve tumors compared to 16 day. NES < -1.4 indicates higher expression in 16 day naïve tumors compared to 8 day.

**Table 3:** Cell cycle regulators are more affected by MT19C at earlier times while translation regulators and ribosomes are affected at later times by GSEA.

Geneset	NES	P-value	FDR
**Treat8 vs. Naive8**			
*Biocarta (n = 184)*			
ATRBRCA pathway	1.74	0.000	0.315
MTA3 pathway	-1.87	0.000	0.141
*GenMapp (n = 107)*			
DNA Replication	1.84	0.000	0.024
G1 to S Cell Cycle Reactome	1.59	0.006	0.122
Krebs TCA Cycle	1.49	0.033	0.219
Galactose metabolism	-1.88	0.000	0.113
Oxidative Phosphorylation	-1.68	0.002	0.139
Nuclear Receptors	-1.59	0.013	0.227
			
*Kegg (n = 165)*			
Cell Cycle	1.94	0.000	0.013
			
*Transcription factor targets (n = 614)*			
E2F1s	1.76-2.05	0.000	0.000-0.008
Bach2	-1.73	0.000	0.037
			
**Treat16 vs. Naive16**			
*Genmapp (n = 107)*			
Ribosomal proteins	2.41	0.000	0.000
Aminoacyl tRNA biosynthesis	2.05	0.002	0.003
Translation Factors	1.66	0.005	0.203

Comparisons are indicated byTreat8 vs. Naive8 and Treat16 vs. Naive 16. NES > 0 indicates higher expression in MT19C treated tumors and NES < 0 indicates higher expression in naïve tumors.

Consistent with the enriched cell cycle pathways, genes regulated by E2Fs were also expressed at higher levels in treated tumors compared to naïve tumors. E2Fs are key transcription factor regulators of the cell cycle, especially the G1-S transition [20]. However, at later times, neither E2F regulated genes nor cell cycle associated genes including CCNE2 were significantly different between the treated and control tumors. This suggests that rapid, initial changes on proliferation and cell cycle regulators are induced by MT19c. Moreover, these observations suggest that tumors collected at different time points reflect specific responses to MT19c.

### PPARγ Activity Enhances SKOV-3 Survival

**Figure 4:**
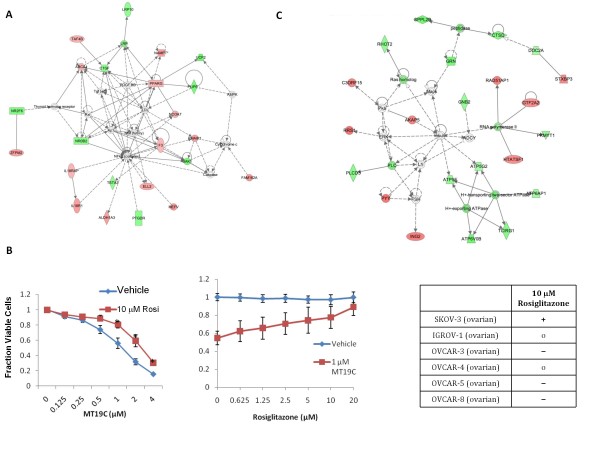
**Rosiglitazone reduces cisplatin and MT19c efficacy in SKOV-3**. **A. PPARγ **network enriched in MT19c treated tumors. Ingenuity Pathway Analysis (IPA) of significantly up and down MT19c (Fold change > 1.4, p < 0.05) regulated genes comparing 16 day treated and naïve tumors identifies a network including PPARγ including PPARγ itself. Red indicates stimulated by MT19c and green indicates down regulation by MT19c. **B**. Addition of 10 μM Rosiglitazone increases the number of viable SKOV-3 cells when treated with MT19c (left panel) in a dose dependent manner (right panel). The number of viable cells was determined by a modified MTT assay using Wst-1. The y-axis represents percent of viable cells normalized to DMSO treated cells. Error bars represent standard deviation. * indicates p < 0.05 from a Student's t-test comparing MT19c treated and Rosiglitazone-MT19c treated cells. 10 μm Rosiglitazone has a range of effects on the NCI-60 panel of six ovarian cancer cell lines. Viability effects of Rosiglitazone in with cisplatin. - indicates < 20% decrease in number viable cells, - - indicates > 20% decrease in number viable cells, + indicates < 20% increase in number viable cells, + + indicates > 20% increase in number viable cells, o indicates no change in number of viable cells. **C**. Insulin centered network identified by IPA. Red indicates stimulated by MT19c and green indicates down regulation by MT19c. Ingenuity Pathway Analysis (IPA) of significantly up and down MT19c (Fold change > 1.4, p < 0.05) regulated genes comparing 16 day treated and naïve tumors identifies a network centered around insulin regulation.

Extrapolating the function of genes from gene expression data is complicated by the observation that genetic background can determine whether a gene is behaving as an oncogene or tumor suppressor [21]. Moreover, multiple interpretations are often consistent with the observed gene expression changes. For example, increased expression from a therapeutic drug may be part of tumor cells' attempt to try to survive the stress or the gene may be directly stimulated to induce cell death. Steroid nuclear receptors are well-known druggable targets that often regulate cell fate [22,23] whose functional role can be rapidly tested with small molecule ligands. We observed that PPARγ itself is significantly stimulated by MT19c. Selecting the top MT19c regulated genes at day 16, (p < 0.05, Fc > 1.4), we observed an extensive network of regulated genes centered around PPARγ in the top scoring network using Ingenuity Pathway Analysis (IPA) (Figure 4A). These data suggest that PPARγ and associated genes were stimulated by MT19c signifying increased PPARγ function. MT19c stimulated genes may be part of the MT19c response or may be part of a survival response as the cells try to stay alive in the presence of a cytotoxic agent. Previous studies suggested that the PPARγ agonist, Rosiglitazone, inhibited ovarian cancer cell growth and enhanced cisplatin therapy [24]. To test PPARγ's role in the chemotherapeutic response of MT19c, we measured the effect of Rosiglitazone on SKOV-3 cell viability when treated in combination with MT19c. Figure 4B shows that Rosiglitazone increases the MT19c IC_50 _from about 2 to 4 μM. We observed similar effects with combinations of Rosiglitazone and cisplatin suggesting that the PPARγ pathway is likely a general survival pathway, and not specific for MT19c activity (Additional File 7, Figure S4). Stimulating PPARγ appears to induce a survival response in SKOV-3 cells suggesting that the observed increased expression of PPARγ is likely an attempt for SKOV-3 cells to survive lethal MT19c activity. We then tested a PPARγ antagonist, GW9662, which did not significantly affect cells as a single agent or in combination with MT19c until very high concentrations (Additional File 7, Figure S4). This suggests that inhibiting PPARγ activity does not necessarily induce cell death. Unlike published studies in other cell lines [24], Rosiglitazone does not appear to significantly affect SKOV-3 proliferation. To test how Rosiglitazone may mediate cisplatin efficacy in other cell lines, we tested combinations of these two molecules in the other NCI60 ovarian cancer cell lines (Figure 4B and Additional File 8, Figure S5). Three cell lines (OVCAR-3, OVCAR-5, and OVCAR-8) showed modest, but reproducible, 20% decreases in cell viability after four days of Rosiglitazone treatment while two others (IGROV-1 and OVCAR-4) showed no significant effects. Thus, SKOV-3 may be a special case, where Rosiglitazone inhibits cisplatin activity and stimulates survival. Like many factors, the genetic background appears to control whether a gene is acting as an oncogene or tumor suppressor [21,25]. These observations highlight how the same factors may lead to opposite functions to control growth and survival in various subtypes of ovarian cancer.

A second IPA identified network, centered on insulin (Figure 4C), is observed when comparing all the naïve and MT19c treated tumors. These observations may be consistent with findings suggesting that IRS 1/2 and ERK 1/2 pathways are down-regulated by MT19c [13]. These expression data along with probing of insulin signaling in cell culture, suggest that MT19c is down-regulating these pro-growth and survival pathways in SKOV-3 as part of its anti-tumor activity.

### MT19c Regulates Genes in Copy Number Aberrations

Copy number changes often drive gene expression of key factors critical for tumor survival and growth. We hypothesized that an antitumor drug such as MT19c may select cells that can evade induced cell death, drive faster growth, and directly affect gene expression through gene dosage effects. We initially observed that GSEA analysis suggested a number of genomic loci were significantly up- and down-regulated by MT19c (Additional Files 9 and 10, Tables S4 and S5). We hypothesized that these MT19c induced changes could be due to the type of tumor cell selected by MT19c compared to naïve tumors. To test this possibility, we performed CGH analysis by purifying DNA from each tumor. DNA was competitively hybridized to Agilent 180 K microarrays with pooled female DNA. For the most degraded treated samples, day 30 xenograft tumors, the microarray signal was poor and was not considered further. The Agilent arrays were segmented using circular binary segmentation (CBS), after outliers greater than four standard deviations from the neighborhood of 20 probes were removed. Each tumor had similar copy number patterns suggesting the same genomes were selected to form the xenografts, with only relatively subtle difference such as in 17 p and 7 p (Additional File 11, Figure S5). Perhaps because of the small number of tumors and the observation that 3/8 MT19c tumors retain some copy loss, no significant trend in expression change for these genes between the treated and naïve tumors is observed.

Copy number can be a major determinant of expression levels for some genes. To determine if gene dosage drives expression of some genes in these xenografts, we mapped segmented CGH probes to each RefSeq gene, to determine a log score reflecting its copy number status. This score was calculated by averaging the log10 copy number ratios of the probes within each gene and selecting transcripts in the top quartile of all expressed genes. A global view of SKOV-3 CNAs show a modest number CNAs compared to other cell lines (Additional File 11, Figure S5) [26].

**Figure 5:**
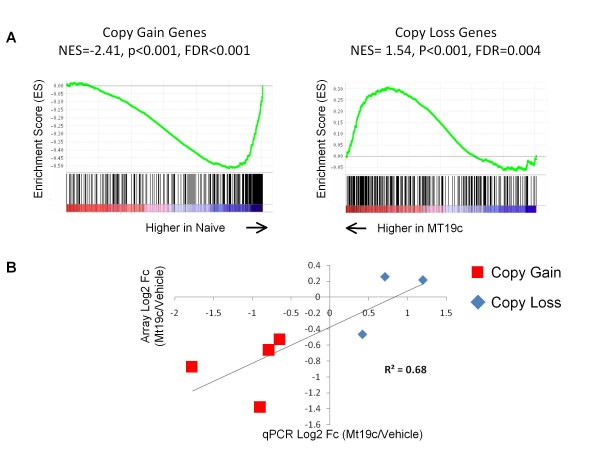
**Genes in copy number gain or loss regions mediate gene expression and are significantly affected by MT19c treatment**. **A**. GSEA Enrichment plots suggest that amplified genes are significantly down-regulated by MT19c compared to naïve tumors while copy number loss genes are significantly up-regulated by MT19c. Significantly amplified (log ratio > 0.3) or deleted genes (log ratio < -0.3) with concomitant high or low expression were selected to define the gene sets. The black lines demonstrate where each gene in the gene set falls within the 15,000 probe sets probing RefSeq transcripts ordered from left to right based on the expression level in MT19c treated or naïve tumors with gene #1 the most highly expressed in MT19c treated tumors. The green line represents the running ES score that becomes more negative as probe sets are identified toward the bottom of the list. More black lines are observed near the top of the rankings in untreated tumors (right side) than in the treated tumors (left side) for amplified genes while genes in LOH regions are biased towards treated tumors (right side). The genes that are most significantly affected by MT19c may be the most important genes mediating SKOV-3 tumor growth and survival. **B**. qPCR of selected genes within CNAs validates microarray observations. Copy loss genes include PBEF1, STEAP1, and ZNF32. Copy gain genes are PNMT, GRN, PAPLN, and SLC25A6.

Because CNAs in tumors are often very large, many genes may be affected by the change in copy number, complicating determination of the functional importance of these genes. We reasoned that genes that are functionally important in CNAs would be differentially affected by an anti-tumor agent. To test if genes in CNAs are differentially regulated by MT19c, we created gene sets including all genes with log ratios > 0.3 in the gene set and expression levels consistent with being regulated by copy number (Additional File 12, Table S6). Using GSEA, these CNA gene sets revealed that MT19c down-regulated amplified genes and up-regulated genes in copy loss regions (Figure 5A), consistent with the idea that many of these genes are likely important for tumor cell growth and survival. qPCR of selected genes suggests the validity of this approach (Figure 5B). For example, a number of genes that may be important for growth and survival are significantly down-regulated by MT19c despite having high copy numbers, including histones (HIST1H3B, HISTH2AE and HIST1H4L), a key enzyme in fatty acid metabolism (DCI), and cell surface proteins possibly important in cell-cell interactions (AGRN, GRN). In particular, the chr17 region including PNMT, ERBB2, and nearby genes such as GRN, is amplified and many of the genes are down-regulated by MT19c. This region is amplified in numerous ovarian cancer cell lines including OVCAR-3, OVCAR-5, OVCAR-8, and CAOV-3 [26] and in a small population of ovarian tumors, although targeting Her2 (ERBB2) has not been successful to date in EOC [27,28]. We observe a number of genes being down-regulated including PNMT, which catalyzes a key step in epinephrine synthesis. Epinephrine may be stimulating tumor growth of ovarian cancer cells, including SKOV-3 [29] and appears to be more sensitive to MT19c action than other genes in this region. These observations suggest that other genes near the ERBB2 locus may also be important for tumor progression and MT19c response.

Many of the copy loss regions span large segments of chromosomes (Additional File 11, Figure S5), significantly complicating the identification of important genes. MT19c up-regulates some genes in copy loss regions (Figure 5). By reducing the number of genes that are expressed and change expression, we have reduced the number of candidates to consider for further experimentation and importance. The up-regulation of these genes when challenged with MT19c, suggests that they may be important to inhibit growth.

## Discussion

Xenograft tumor model systems are powerful tools for the evaluation and development of anti-tumor therapeutics. These models are especially useful for ovarian cancer, where only limited mouse models have been developed [30]. SKOV-3 ovarian cancer cells are one of the more commonly used cell lines to model ovarian cancer. We aimed to understand what may drive xenograft tumor growth, which likely differs from growth in cell culture conditions, and what factors may be important for anti-tumor treatment. As a model, we tested the effects of MT19c, a vitamin D derivative that shows promising pre-clinical properties as demonstrated here and elsewhere [6,15,16]. We used multiple approaches to identify significantly MT19c regulated genes, pathways and networks with experimental support suggesting the functional importance of the insulin and PPARγ networks for MT19c efficacy. In particular, we found that PPARγ and PPARγ-controlled networks are up-regulated in treated tumors and stimulation of PPARγ with Rosiglitazone inhibited the chemotherapeutic efficacy in SKOV-3 cells. Finally, we propose an approach integrating copy number and expression data to identify which genes within CNAs are most likely to be important for tumor progression and chemotherapy. We propose that genes with high or low copy number, along with significant gene expression changes in response to an anti-tumor agent indicate genes important tumorigenesis.

This approach of linking copy number and drug induced expression changes may be a viable approach to identify particularly important genes for tumor progression. Although the majority of high copy number and high expression genes were affected by MT19c, many were not. A few high copy number genes such as RPL23 and RPS29 were significantly stimulated by MT19c. These high copy number genes may be up-regulated to help SKOV-3 cells survive in response to a lethal compound such as MT19c. But, their up-regulation upon MT19c treatment suggests that their high expression and high copy number may be serving a different role than down-regulated genes. Combining gene expression and copy number can reduce the number of genes to consider for further study. These data also suggest that genes regulated by dosage play an important role in cancer cells' response to chemotherapy. Together, these data provide insights into general pathways important for tumor progression and survival as well as MT19c efficacy.

Our observations suggest that in some cases the PPARγ network is stimulated to help ovarian cancer cells survive as suggested by Rosiglitazone treatment increasing the IC_50_s of MT19c and cisplatin. The observations suggest that stimulation of PPARγ by Rosiglitazone increases SKOV-3 chemotherapy resistance (Figure 4B). Rosiglitazone has been reported to inhibit growth and enhance cisplatin efficacy in some ovarian cancer cell lines, though SKOV-3 was not tested [24]. These findings suggest that additional study is warranted to understand the conditions in which Rosiglitazone may be an effective chemotherapeutic and when it may actually promote survival of ovarian cancer cells.

We aimed to understand which gene expression networks may be important for SKOV-3 xenograft tumors progression and MT19c response. We found that significantly different conclusions may be made depending on how long the tumor was treated before the specimen was collected. Because xenograft tumors, much like patient tumors, are continuously evolving during growth, drug responses may differ at each captured state of the tumor at the time the specimen is collected for analysis. Genes involved in the cell cycle, energy metabolism, and DNA replication machinery are significantly affected by MT19c at the earlier, day 8 time point, while regulation of protein synthesis and ribosomes were significantly up-regulated at the later day 16 time point. No significant enrichment of cell cycle and DNA replication machinery was observed after 16 days of MT19c treatment. These pathways are often observed to be affected in tumors as control of metabolism and the cell cycle are often critical for tumor growth and survival. From gene expression data, it is difficult to conclude whether the effects are direct or indirect. These data identify candidate genes to test for their importance in mediating chemosensitivity in ovarian cancer cells as well as possible specific factors related to MT19c that can be discriminated with further study.

A significant contribution to these differences appears to originate from changes in the tumor as it progresses. This is highlighted when comparing the naïve tumors at the day 8 and 16 time points. When comparing the day 8 and 16 naïve tumors, cell cycle and DNA replication machinery is expressed higher at later times. We then observed that MT19c down-regulates the cell cycle at early times and yet not at later times when many of these genes are expressed at higher levels. Many of these genes are controlled by E2F. These observations suggest that control of the cell cycle depends on when the tumor is collected. In patients, the exact place in tumor progression/growth is always different and thus this alone can explain expression differences, as opposed to the inherent character of genomic differences of the tumor. Among many other factors, these expression changes during tumor growth may be inherent to tumors and could be a source of heterogeneity in patient samples. In probing xenograft tumors, these observations highlight the importance of examining changes at multiple time points, and not just at an arbitrarily defined endpoint.

Integrating copy number and expression measurements has proven valuable to gain insight into tumor networks and regulation [31]. However, determining which genes are drivers of tumorigenesis and which may be passengers remains a challenging problem, typically requiring extensive experiments to test the function of identified factors [32]. Copy number can be a mechanism driving gene expression levels. Typically this is assessed by correlating the measured expression and copy number values. Here, we propose an extension of the copy number and expression comparison, in which we identify important genes in CNAs by examining changes driven by an anti-tumor therapy such as MT19c. Alternatively, these expression changes could also indicate chromatin deregulation of these genes, suggesting that the observed differential regulation is simply a result of chance. The many loci differentially regulated by MT19c suggest extensive changes in epigenetic control by MT19c. We believe the simplest explanation is that the MT19c induced down-regulation strongly suggests that at least some of these amplified genes are important for growth.

Together, these observations suggest that some of the amplified genes are important for tumorigenesis consistent with the hypothesis that amplified genes are selected for tumor growth and survival. Similarly, genes at lower gene dosage are stimulated by MT19c to induce cell death or at least limit growth. These observations suggest that a significant fraction of these CNA genes are important for tumor growth and survival. We speculate that these loci are being deregulated epigenetically by chromatin changes to overcome the gene dosage determined by the DNA copy number at the time points tested. Thus, the combination of DNA copy number and the mRNA expression behavior in response to MT19c suggest that these genes may be among the most important for SKOV-3 xenograft progression.

MT19c has strong anti-tumor effects in multiple ovarian cancer cell lines including SKOV-3; however, the mechanism of MT19c action remains unclear. We observed many factors and pathways affected by MT19c, including some related to tumor growth and survival, such as DNA damage response, apopotic genes, insulin, and PPARγ. Similar to PPARγ, it is likely that many of these factors are general survival and pathway factors for SKOV-3 cells. Identifying specific mechanisms from expression data based on small molecule perturbations remains challenging, and requires significant additional functional studies. These data highlight how MT19c affects specific pathways, such as PPARγ and insulin signaling, that can be tested in additional cell lines and in vivo models to determine their importance. These studies could lead to the development of biomarkers to help determine which features effect MT19c efficacy in pre-clinical and clinical models.

Combining copy number and changes in gene expression approach in multiple cell lines may prove useful to identify particularly important genes in mediating survival and drug responses in ovarian cancer. The major copy number changes in the SKOV3 xenografts are observed in the SKOV-3 cell line as reported [26] including the amplifications on chromosomes 3 and 17 and LOH on chromosome 1.

## Conclusions

In summary, these data characterize SKOV-3 ovarian xenograft tumors and identify candidate factors important for tumor progression and response to chemotherapy. We have characterized the time dependent expression changes of SKOV-3 xenograft tumors in response to a novel chemotherapeutic. We also identify a possible role for PPARγ and Rosiglitazone stimulation in mediating chemotherapy in some ovarian cancer cells. Further study examining the widely varying functions of PPARγ in different genetic and epigenetic backgrounds is warranted. Finally, we propose a new integrated genomics approach that combines copy number and expression data to identify candidate tumor drivers. We find a general bias for down-regulation of amplified genes by the cytotoxic agent, MT19c, highlighted by strong down-regulation of genes, such as PNMT, in the same amplicon as the ERBB2 locus. These observations suggest that at least some genes regulated by dosage are critical for chemotherapy responses. Together, these data suggest that integrated genomics can provide important insights into the behavior of xenograft tumor systems, which are commonly used to evaluate tumor progression and drug efficacy.

## List of Abbreviations used

The abbreviations used are: EOC: epithelial ovarian cancer; PPARg: peroxisome proliferator-activated receptorγ; VDR: vitamin D receptor; CNA: copy number alteration; H&E: hematoxylin and eosin; CCNE2: cyclin E2; CGH: Comparative Genomic Hybridization.

## Competing interests

The authors declare that they have no competing interests.

## Authors' contributions

Conception and design: LB and ASB, Animal studies: AS and KKK, MT19c synthesis and verification of efficacy: RS, Nucleic acid isolation and in vitro studies: AS and DHM, Data analysis and interpretation: ASB, LB, SH, AR, and BJR, Manuscript writing: ASB and LB, Final approval of manuscript: AS, AF, DHM, SH, KKK, AR, RKS, BJR, LB, ASB.

## Pre-publication history

The pre-publication history for this paper can be accessed here:

http://www.biomedcentral.com/1471-2407/11/308/prepub

## Supplementary Material

Additional file 1 **Table S1**. List of primers used for real-time qPCR Click here for file

Additional file 2 **Figure S1**. VDR is expressed in SKOV-3. Immunoblot showing VDR is expressed in SKOV-3 and VDR levels do not change in response to MT19c. Click here for file

Additional file 3 **Figure S2**. Immunohistochemistry suggests tumors are majority tumor cells with little invasion. **A**. H&E staining of naïve tumor. **B**. CA-125 staining of a representative naïve tumor. **C**. CA-125 staining of a MT19C treated tumor. Click here for file

Additional file 4 **Table S2**. List of genes down-regulated by MT19c. Click here for file

Additional file 5 **Table S3**. List of genes up-regulated by MT19c. Click here for file

Additional file 6 **Figure S3**. qPCR and microarray data correlate. Pooling samples from the 16 day MT19C treated and naïve tumors indicates that qPCR and microarray data agree on the direction of the fold change upon MT19C treatment. Error bars are standard deviation. Panels A, B, and C indicate fold range relative to calnexin and tumor N8A. Panel D calculates the fold change of the array and qPCR data for the three genes indicated. ABCA1 and PPARγ were considered significant in the microarray data. Click here for file

Additional file 7 **Figure S4**. PPARγ signaling mediates chemosensitivity. **A**. Rosiglitazone also inhibits cisplatin in SKOV-3. Increasing concentration of Rosiglitazone show a dose dependent increase in the number of cells with cisplatin similar to the dose dependent effects observed with MT19c. **B**. The PPAR γ antagonist, GW9662, shows no significant effect on MT19c efficacy in a dose response curve. Click here for file

Additional file 8 **Figure S5**. Dose response curves of cisplatin(CDDP) in the presence of Rosiglitazone (Rosi) in the six NCI-60 ovarian cancer cell lines. The number of viable cells was determined by Wst-1 after four days. Rosi inhibits CDDP in some lines (SKOV-3) while in others (OVCAR-3 and OVAR-5) Rosi supports CDDP induced cell death. Click here for file

Additional file 9 **Table S4**. GSEA data of chromosome loci enriched in MT19c treated tumors. Click here for file

Additional file 10 **Table S5**. GSEA data of chromosome loci enriched in naive tumors Click here for file

Additional file 11 **Figure S6**. Global copy number views of SKOV3 xenografts. Heat maps of all > 2 fold copy number changes from Agilent 180 K CGH microarrays compared to pooled normal female DNA. Notice the amplification on chromosome 17 which includes ERBB2. The genome has relatively few aberrations compared to other ovarian cancer cell lines and many ovarian tumors. Red indicates a copy number gain and blue indicates a copy number loss. Click here for file

Additional file 12 **Table S6**. List of genes used to define the copy number aberration gene set. Click here for file
