# Supplementary figures and images for: Integrated genomics of ovarian xenograft tumor progression and chemotherapy response
Source: BMC Cancer. 2011 Jul 22;11:308. doi: 10.1186/1471-2407-11-308 (PMC3155912; doi:10.1186/1471-2407-11-308)

## Slide 1
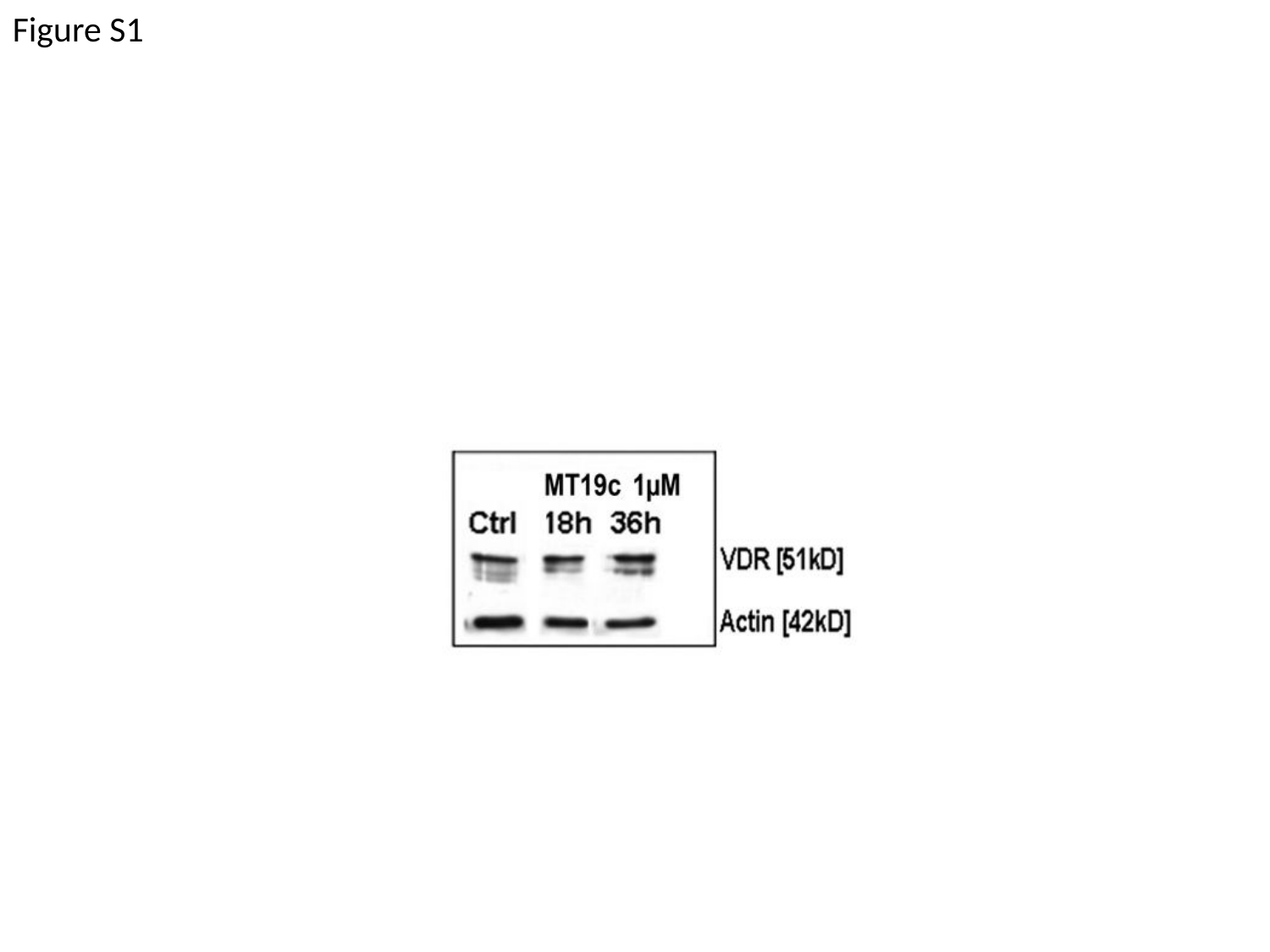

Figure S1

Supplement: Additional file 2 — Figure S1. VDR is expressed in SKOV-3. Immunoblot showing VDR is expressed in SKOV-3 and VDR levels do not change in response to MT19c. [file 1471-2407-11-308-S2.PPT]

## Slide 1
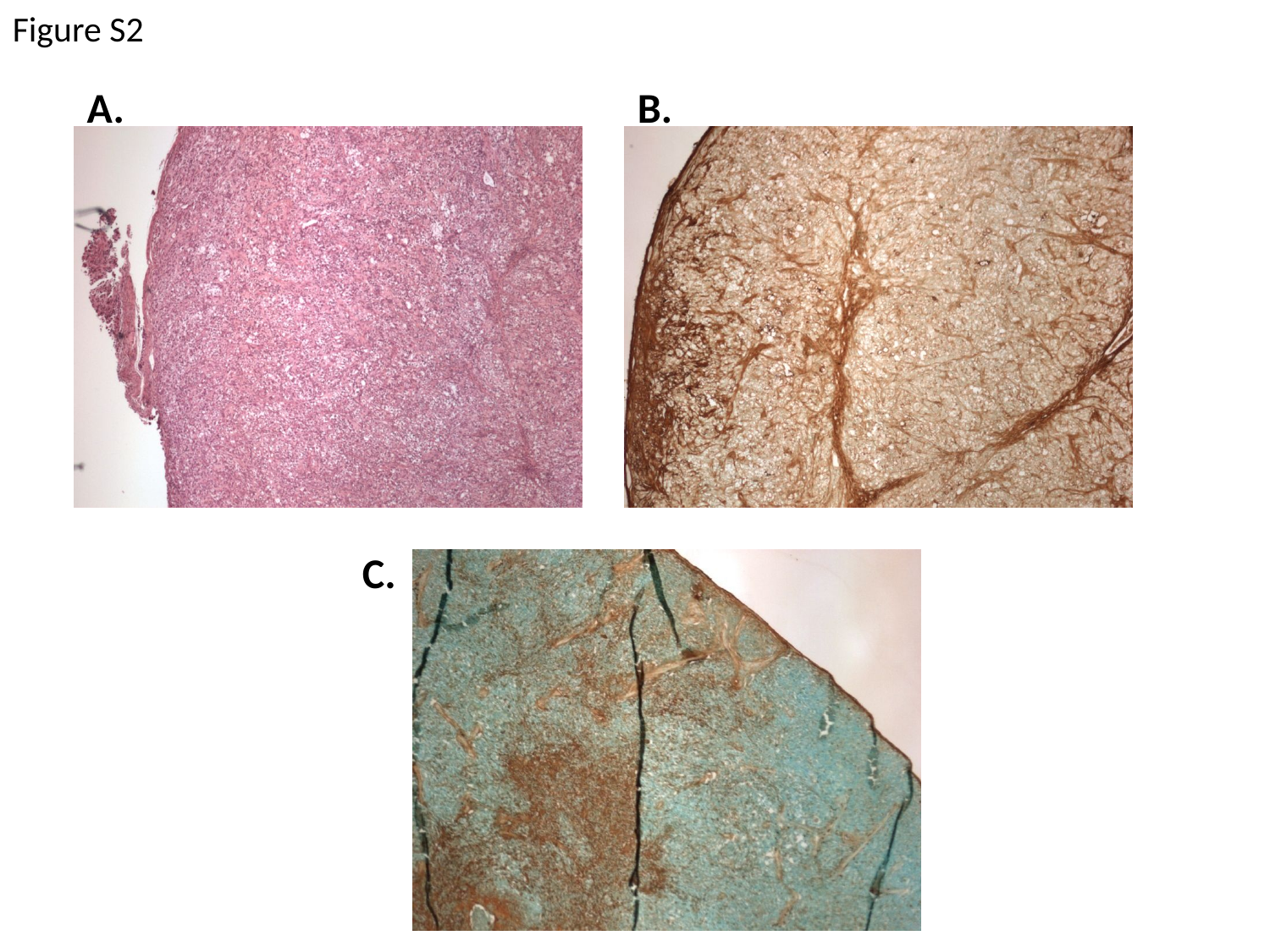

Figure S2
A.
B.
C.

Supplement: Additional file 3 — Figure S2. Immunohistochemistry suggests tumors are majority tumor cells with little invasion. A. H&E staining of naïve tumor. B. CA-125 staining of a representative naïve tumor. C. CA-125 staining of a MT19C treated tumor. [file 1471-2407-11-308-S3.PPT]

## Slide 1
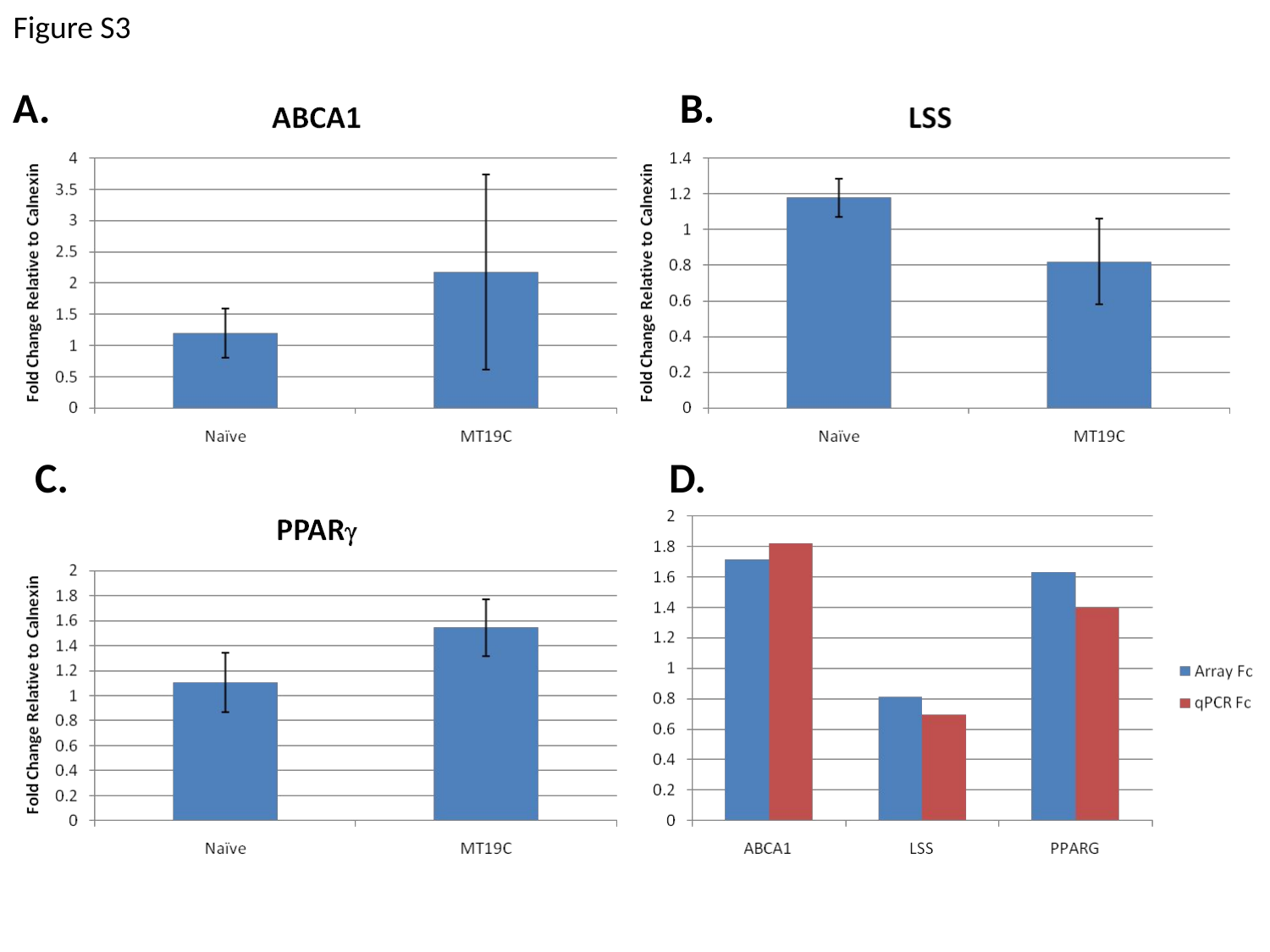

Figure S3
A.
B.
C.
D.

Supplement: Additional file 6 — Figure S3. qPCR and microarray data correlate. Pooling samples from the 16 day MT19C treated and naïve tumors indicates that qPCR and microarray data agree on the direction of the fold change upon MT19C treatment. Error bars are standard deviation. Panels A, B, and C indicate fold range relative to calnexin and tumor N8A. Panel D calculates the fold change of the array and qPCR data for the three genes indicated. ABCA1 and PPARγ were considered significant in the microarray data. [file 1471-2407-11-308-S6.PPT]

## Slide 1
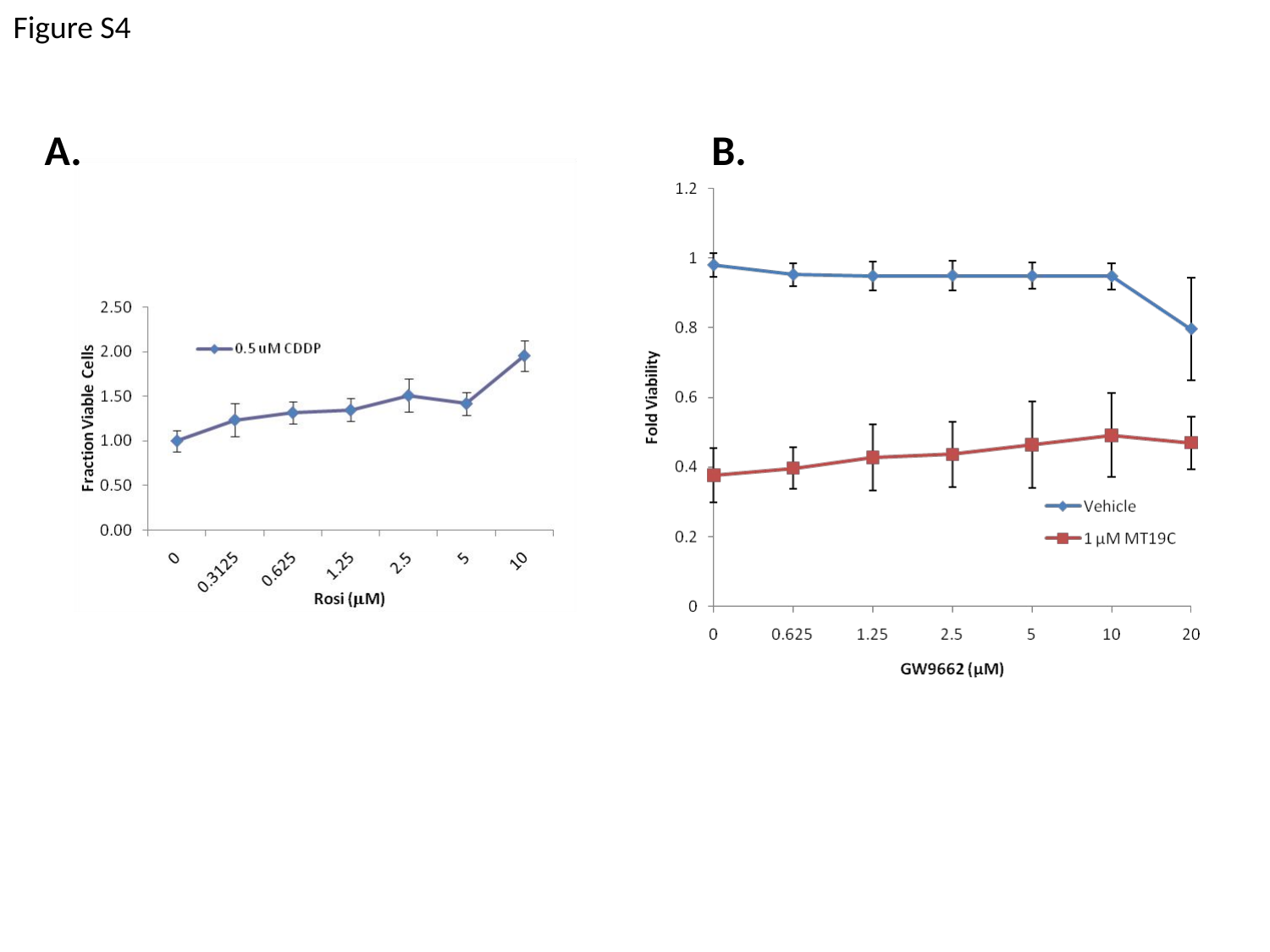

Figure S4
A.
B.

Supplement: Additional file 7 — Figure S4. PPARγ signaling mediates chemosensitivity. A. Rosiglitazone also inhibits cisplatin in SKOV-3. Increasing concentration of Rosiglitazone show a dose dependent increase in the number of cells with cisplatin similar to the dose dependent effects observed with MT19c. B. The PPAR γ antagonist, GW9662, shows no significant effect on MT19c efficacy in a dose response curve. [file 1471-2407-11-308-S7.PPT]

## Slide 1
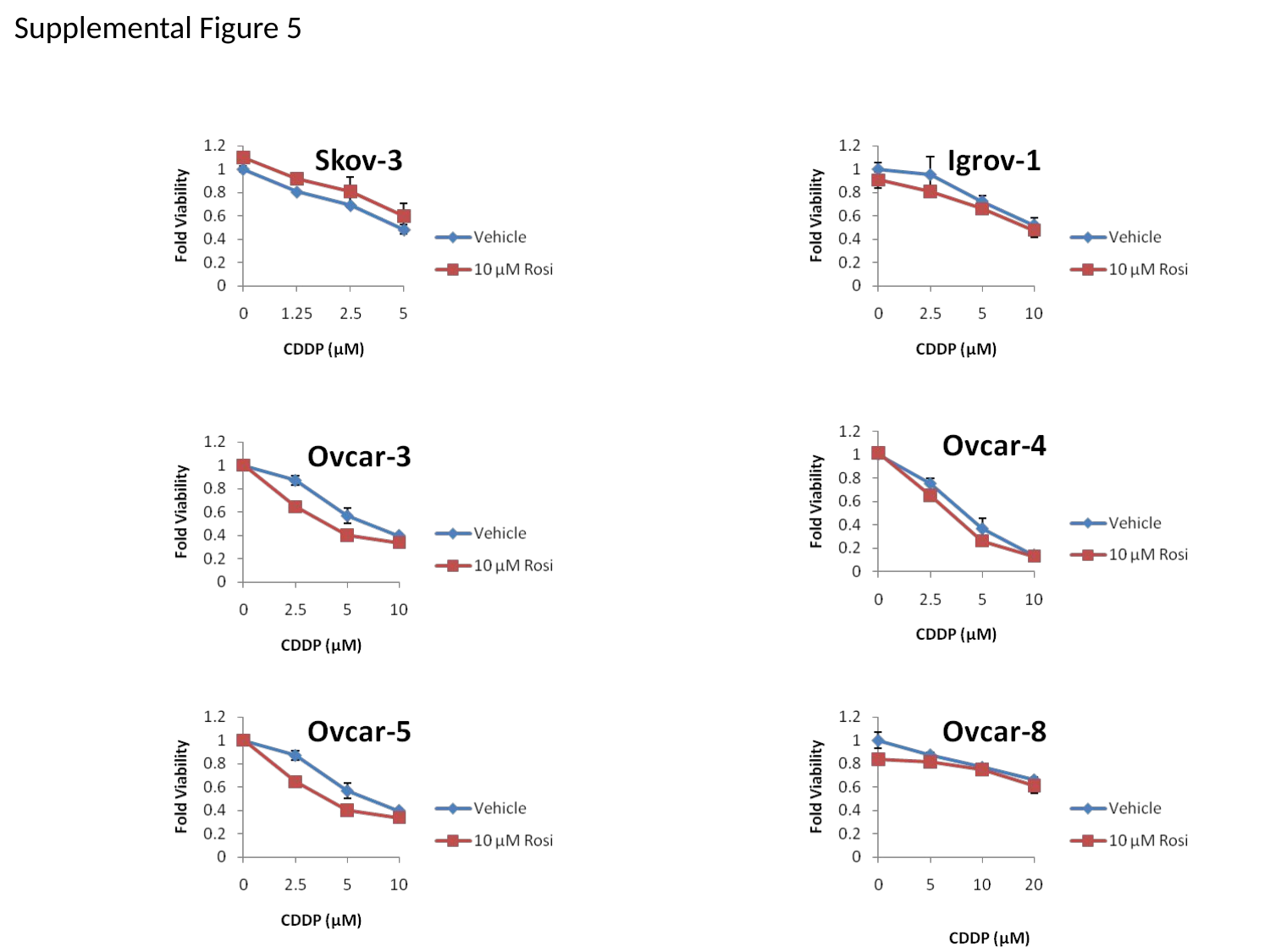

Supplemental Figure 5

Supplement: Additional file 8 — Figure S5. Dose response curves of cisplatin(CDDP) in the presence of Rosiglitazone (Rosi) in the six NCI-60 ovarian cancer cell lines. The number of viable cells was determined by Wst-1 after four days. Rosi inhibits CDDP in some lines (SKOV-3) while in others (OVCAR-3 and OVAR-5) Rosi supports CDDP induced cell death. [file 1471-2407-11-308-S8.PPT]

## Slide 1
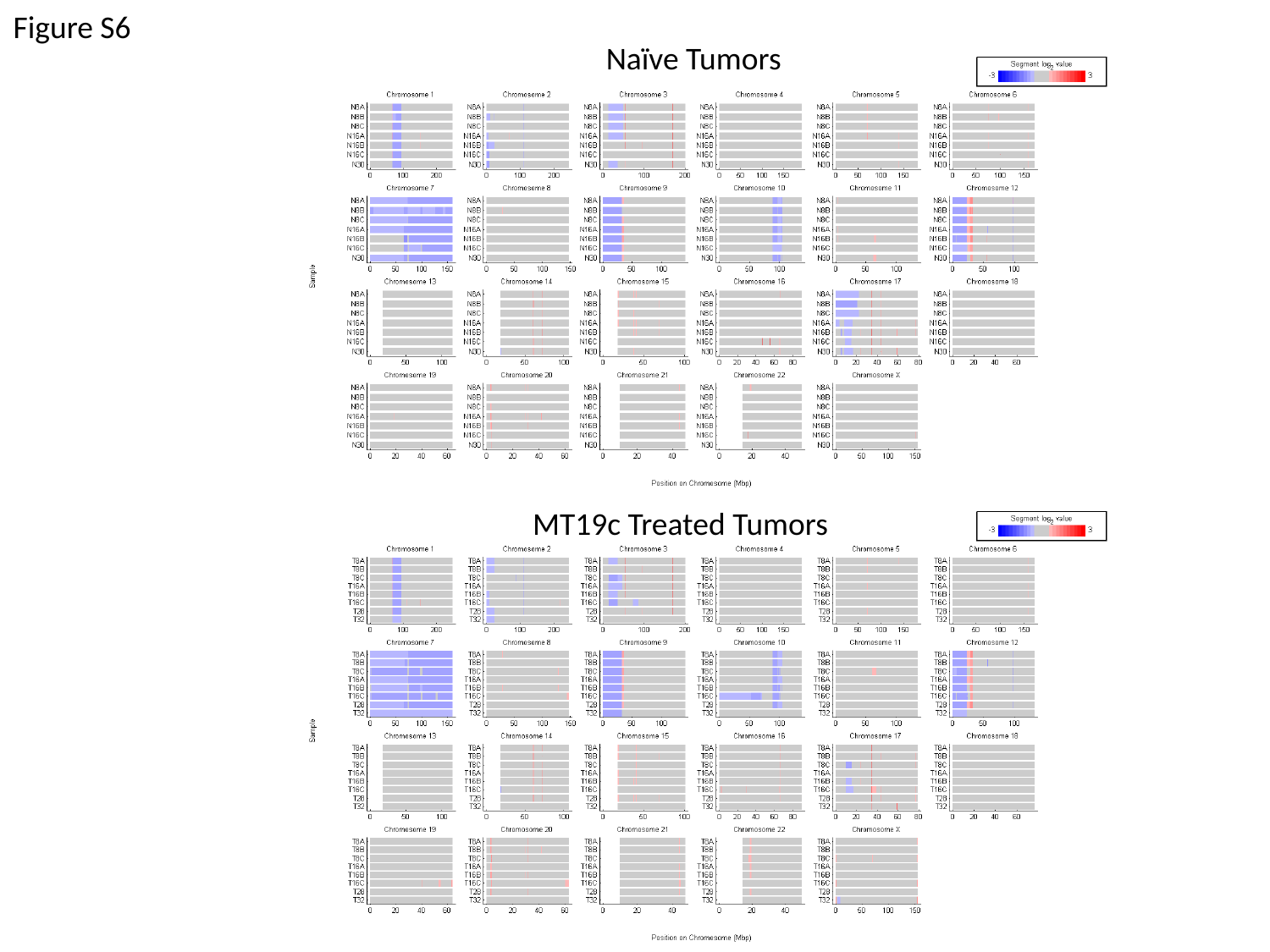

Figure S6
Naïve Tumors
MT19c Treated Tumors

Supplement: Additional file 11 — Figure S6. Global copy number views of SKOV3 xenografts. Heat maps of all > 2 fold copy number changes from Agilent 180 K CGH microarrays compared to pooled normal female DNA. Notice the amplification on chromosome 17 which includes ERBB2. The genome has relatively few aberrations compared to other ovarian cancer cell lines and many ovarian tumors. Red indicates a copy number gain and blue indicates a copy number loss. [file 1471-2407-11-308-S11.PPT]
